# Resting-state abnormalities in Autism Spectrum Disorders: A meta-analysis

**DOI:** 10.1038/s41598-019-40427-7

**Published:** 2019-03-07

**Authors:** Way K. W. Lau, Mei-Kei Leung, Benson W. M. Lau

**Affiliations:** 1 Department of Special Education and Counselling, The Education University of Hong Kong, Hong Kong, China; 2 Department of Rehabilitation Sciences, The Hong Kong Polytechnic University, Hong Kong, China

## Abstract

The gold standard for clinical assessment of Autism Spectrum Disorders (ASD) relies on assessing behavior via semi-structured play-based interviews and parent interviews. Although these methods show good sensitivity and specificity in diagnosing ASD cases, behavioral assessments alone may hinder the identification of asymptomatic at-risk group. Resting-state functional magnetic resonance imaging (rs-fMRI) could be an appropriate approach to produce objective neural markers to supplement behavioral assessments due to its non-invasive and task-free nature. Previous neuroimaging studies reported inconsistent resting-state abnormalities in ASD, which may be explained by small sample sizes and phenotypic heterogeneity in ASD subjects, and/or the use of different analytical methods across studies. The current study aims to investigate the local resting-state abnormalities of ASD regardless of subject age, IQ, gender, disease severity and methodological differences, using activation likelihood estimation (ALE). MEDLINE/PubMed databases were searched for whole-brain rs-fMRI studies on ASD published until Feb 2018. Eight experiments involving 424 subjects were included in the ALE meta-analysis. We demonstrate two ASD-related resting-state findings: local underconnectivity in the dorsal posterior cingulate cortex (PCC) and in the right medial paracentral lobule. This study contributes to uncovering a consistent pattern of resting-state local abnormalities that may serve as potential neurobiological markers for ASD.

## Introduction

Autism spectrum disorder (ASD) is a complex neurodevelopmental disorder that is characterized by persistent social and communication deficits, and restricted and repetitive behaviors and interests. A wide range of genetic and developmentally early environmental factors play an essential role in the etiological heterogeneity in ASD^1,2^. In general, the worldwide population prevalence of ASD is about 1%^3,4^, with a trend of increasing prevalence of ASD across different countries^5,6^. Longitudinal studies indicate that only 20% of individuals with ASD seem to obtain a good adult outcome as indicated by the quality of independent living, friendships and participation in employment^7^. The high prevalence and poor prognosis of ASD result in an enormous cost for society and for the individual, in loss of productive years and cost of educational support^8^. Early intervention is needed to decelerate, or even prevent, the developmental cascade that manifests in the disorder. To do so, an accurate and early diagnosis of ASD is essential.

To date, the gold standard for clinical assessment of ASD includes administration of the Autism Diagnostic Observation Schedule 2 (ADOS-2)^9^ and the Autism Diagnostic Interview-Revised (ADI-R)^10^ that assess behavior by semi-structured play-based interviews and parent interviews, respectively. Although the combination of ADOS and ADI-R has been found to have good sensitivity and specificity in diagnosing ASD cases as young as 12 months old, the predictive validity of the measure can be markedly reduced if the examiner who carries out the test is not the primary diagnostician^11^. Furthermore, this kind of behavioral assessments require well-trained examiner to carry out, yet training can be time-consuming, expensive and difficult to procure^11^. More importantly, children comorbid with ASD and attention-deficit/hyperactivity disorder (ADHD), which is not uncommon, could delay ASD diagnosis due to some shared symptoms between ASD and ADHD in 2–3 year old. In a large population-based study, the presence of ADHD and/or sensory processing symptoms in children with ASD delayed an autism diagnosis for around 3 years^12^. These add to the difficulty of admitting clinical cases into the right intervention program before school age, which significantly reduces the effectiveness of behavioral therapies because the most sensitive window for neural and behavioral changes has been missed^13,14^. In addition, many infants who were eventually diagnosed with ASD remained asymptomatic at 6–9 months old^15^. It is difficult to use behavioral measures alone to identify at-risk group at an early life stage during which crucial developmental delays may be manifesting. The development of objective biomarkers is, therefore, essential for supplementing traditional behavioral measures for better diagnosis of ASD. To the best of our knowledge, there is currently no objective biomarkers exist for diagnosing ASD.

In the past decades, researchers attempted to find out reliable biomarkers to predict ASD by understanding its etiology and pathology. For instance, de novo missense, promoter, and enhancer mutations were found in autism probands compared to the unaffected siblings in 516 idiopathic autism families. Furthermore, in the same studied cohort, oligogenic de novo mutations were enriched for expression in striatal neurons in autism probands^16^, suggesting a complex genomic pattern in ASD. In addition, epigenetic changes through DNA methylation and trans-sulfuration were also noted in ASD cases, indicating interaction effects between environmental and genetic factors on ASD^17^. On the other hand, there is evidence supporting the presence of proteomic biomarkers in predicting ASD such as increased glycation endproducts^18^. Another line of research suggests that autism could be associated with the diversity of intestinal microflora. For instance, the presence of autistic symptoms was found to be associated with less diverse gut microbiomes in 20 autistic children^19^. Functional studies are required to confirm the role of those mutated genes in ASD using animal models. More future studies in human subjects are also warranted to investigate the sensitivity and specificity of these potential biomarkers in predicting ASD.

There is a growing number of studies using functional magnetic resonance imaging (fMRI) approach to study the neural underpinnings of behaviors associated with ASD. In the past decades, a large number of task-based fMRI studies were conducted, and meta-analyses on these task-based fMRI studies were also available. For instance, Philip and colleagues conducted a systematic review and meta-analysis on task-based fMRI studies of ASD including motor, visual processing, executive function, auditory and language, basic social processing and complex social cognition tasks^20^. They concluded that the most reliable finding was a disturbance to the function of social brain regions, whereas comparisons on other tasks were difficult due to the diversity and complexity of tasks used across studies. Such limitations together with the fact that task difficulty may preclude some children, especially infants, from participation, make task-based fMRI features not to be ideal as biomarkers for ASD^21^.

Resting-state fMRI (rs-fMRI) is a promising alternative to the study of large-scale organization of the typically and atypically developing brain in infants and toddlers^22^. rs-fMRI data can be acquired in 5–6 minutes as the participants lay in the MRI scanner with either eyes closed or fixated on a cross-hair. It helps reveal the coupling of functional brain networks independent of task performance. These advantages make it particularly suitable for examining brain maturation in pediatric and clinical populations such as ASD participants who often have a wide range of functioning levels. Numerous studies have attempted to work out local resting-state differences between subjects with ASD and typically developing (TD) age-matched controls. However, the findings have been inconsistent. For example, during resting state, Maximo and colleagues^23^ observed local overconnectivity in posterior temporal regions, whereas Paakki and colleagues^24^ found reduced local synchronization in superior temporal region, in subjects with ASD compared to TD controls. Also, some studies observed local overconnectivity in the frontal, temporal and occipital lobes in subjects with ASD^23,25,26^ but some other studies did not detect any local overconnectivity in subjects with ASD^27–31^. Small sample sizes, phenotypic heterogeneity in ASD subjects and/or the use of different analytical methods across studies may have contributed to the inconsistencies. Although the Autism Brain Imaging Data Exchange (ABIDE) initiative could provide a large dataset to avoid false-negative errors, many studies that utilized ABIDE dataset adopted a cluster-wise inference approach in their statistical analyses. It has recently been suggested that the use of cluster-wise inference in parametric statistical methods may lead to inflated false-positive rates, especially when a lenient cluster-defining threshold (CDT) such as uncorrected p = 0.01 was used^32^. A systematic and quantitative meta-analysis is, therefore, timely to unearth consistent local resting-state abnormalities that are truly unique to ASD regardless of all methodological differences. However, a meta-analysis of the resting-state abnormalities in subjects with ASD has yet to be performed.

The current study aims to investigate the neural abnormalities in local resting-state connectivity of ASD regardless of subject age, IQ, gender and disease severity, using activation likelihood estimation (ALE). Any consistent pattern that could be identified from a diverse sampling should represent the core resting-state abnormalities that are sensitive to ASD. Findings from this ALE meta-analysis study could provide insight into the development of neural biomarkers for diagnosis of ASD in future studies.

## Results

Two-hundred and four ASD subjects (175 males) with mean age of 20.30 ± 10.20 and 220 TD subjects (185 males) with mean age of 19.87 ± 9.72 were included. All subjects had an IQ higher than 80 (Table 1).

**Table 1:** Subjects demographic in included studies.

Study	Method of analysis	N	Age in years (SD)	Gender (M:F)	IQ (SD)	Diagnostic criteria	Contrasts	Foci
Paakki *et al*.^24^	ReHo	28 ASD	14.58 (1.62)	20:8	>80	ADI-R ADOS ICD-10	TD > ASD	6
		27 TD	14.49 (1.51)	18:9			ASD > TD	4
von dem Hagen *et al*.^27^	ICA	15 ASD	30 (8)	15:0	116 (12)	ADI-R ADOS	TD > ASD	1
		24 TD	25 (6)	24:0	118 (13)		ASD > TD	N.S.
^a^Mueller *et al*.^28^	ICA	12 ASD	35.5 (11.4)	9:3	111.3 (13.4)	ICD-10	TD > ASD	2
		12 TD	33.3 (9.0)	8:4	110.8 (14.4)		ASD > TD	N.S.
^b^Maximo *et al*.^23^	ReHo	29 ASD	13.8 (2.4)	25:4	107.9 (19.0)	ADOS ADI-R	TD > ASD	7
		29 TD	13.5 (2.2)	22:7	108 (8.9)		ASD > TD	4
^c^Bos *et al*.^29^	ICA	27 ASD	11.8 (1.9)	27:0	114.0 (14.4)	DSM-IV	TD > ASD	3
		29 TD	12.2 (2.1)	29:0	113.9 (15.0)		ASD > TD	N.S.
Itahashi *et al*.^30^	fALFF	50 ASD	30.82 (7.39)	43:7	105.6 (14.12)	DSM-IV	TD > ASD	4
		50 TD	31.6 (7.6)	43:7	108.09 (8.98)		ASD > TD	N.S.
Jann *et al*.^25^	ASL	17 ASD	13.8 (2.0)	13:4	107.8 (18.7)	ADOS, ADI-R	TD > ASD	1
		22 TD	12.8 (3.6)	19:3	107.8 (14.3)		ASD > TD	6
Nair *et al*.^26^	ReHo	26 ASD	13.93 (2.43)	23:3	106.04 (18.47)	ADOS, ADI-R	TD > ASD	4^d^/5^e^/3^f^
		27 TD	13.83 (2.26)	22:5	106.89 (17.19)		ASD > TD	4^d^/3^e^/5^f^

Abbreviations: ADI-R, Autism Diagnostic Interview-Revised; ADOS, Autism Diagnostic Observation Schedule; ASD, Autism Spectrum Disorder; ASL, Arterial Spin Labeling; DSM, Diagnostic and Statistical Manual of Mental Disorders; fALFF, Fractional Amplitude of Low Frequency Fluctuations; ICA, Independent Component Analysis; ICD-10, International Statistical Classification of Diseases and Related Health Problems, Tenth Edition; N.S., No significant group difference; ReHo, Regional Homogeneity; SD, Standard deviation; TD, Typical Developing Control. ^a^Only results with corrected *p* < 0.05 were included. ^b^Only results of ReHo (27 voxels) were included. ^c^Authors of the study were contacted by email for the missing coordinates of the left middle temporal gyrus. ^d^Results of standardized ReHo with the use of global signal regression (GSR). ^e^Results of standardized ReHo without using GSR. ^f^Results of nonstandardized ReHo without using GSR.

**Figure 1:**
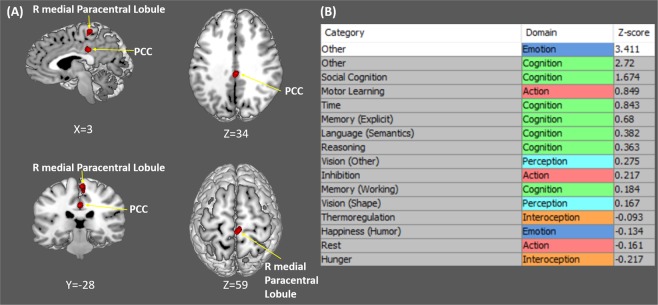
(**A**) Reduced resting-state local connectivity in subjects with ASD compared to typically developing participants, including the right medial paracentral lobule and the dorsal posterior cingulate cortex (PCC) cluster. (**B**) Results of the Behavioral Analysis of the dorsal PCC cluster. The dorsal PCC cluster was found to be engaged in a sub-domain of Emotion called ‘other’ (Z-score = 3.411). R = Right.

For the ASD < TD comparison, all the three ALE analyses consistently found two significant clusters in the right medial paracentral lobule (Brodmann area, BA, 5) and dorsal posterior cingulate cortex (PCC) (BA 31). According to the Vogt’s model^33,34^, the PCC consists of the dorsal and ventral parts, which are superior and posterior to the splenium of the corpus callosum, respectively. As shown in Fig. 1A, our dorsal PCC cluster is situated superior to the splenium of the corpus callosum. Based on the ALE output, the right medial paracentral lobule cluster was contributed by foci from three studies^23,26,28^, and the dorsal PCC cluster was contributed by foci from two studies^23,26^.

For the ASD > TD comparison, no significant clusters were found. For both comparisons, all results were basically the same no matter which set of Nair *et al*.’s results^26^ was included in the ALE analyses. The corresponding anatomical locations and peak ALE maxima are shown in Fig. 1A and Table 2.

**Table 2:** Resting-state abnormalities in subjects with ASD compared to TD controls.

	Brain region	BA	Coordinates (MNI space)			Volume (mm^3^)	Extrema Value
			x	y	z		
ASD < TD	Posterior cingulate cortex	31	2	−28	34	456	0.01525
	Paracentral lobule	5	4	−30	60	600	0.01376
ASD > TD	*No significant findings*						

Abbreviations: ASD, Autism Spectrum Disorder; BA, Brodmann Area; MNI, Montreal Neurological Institute; TD, Typically Developing Control.

To further examine the functional role of the right medial paracentral lobule and dorsal PCC, behavioral profiling was performed using the Behavioral Analysis plugin (v2.2) via the Mango software (http://ric.uthscsa.edu/mango/). The behavioral domain meta-data of the BrainMap database, which has five major domains (Action, Cognition, Emotion, Interoception, and Perception) and fifty-one sub-domains of mental operations^35^, was queried to characterize the behavioral domain specificity of the two resultant brain regions. Z-scores were calculated for all fifty-one sub-domains, and a Z-score > 3.0 is regarded as statistically significant. According to the behavioral domain meta-data of the BrainMap database, the dorsal PCC cluster was found to be engaged in a sub-domain of Emotion called ‘other’ (Z-score = 3.411) (Fig. 1B), which is defined as ‘*any affective processes that qualify as Emotion*, *but do not fit into any of the other Emotion sub-domains*’. Alternatively, the right medial paracentral lobule was not found to be engaged in any of the domains/sub-domains (all Z-scores < 3.0).

## Discussion

In this ALE study, we observed consistently lower resting-state local connectivity in the dorsal PCC and right medial paracentral lobule in subjects with ASD compared to TD controls across eight different studies that included subjects with mixed characteristics. No ASD-related local overconnectivity was found. These results remain the same no matter which set of result from Nair *et al*.’s study^26^ was included in the ALE analyses. This indicates the stability of our results regardless of the GSR and/or standardization step performed in one of the contributing studies. These support our aim of identifying a consistent neural pattern underlying ASD regardless of subject variations and methodological differences.

Cluster-level FWE correction method was applied in this ALE meta-analysis. Although cluster-wise inference may have a higher chance of inflating false-positive rates (especially when a lenient CDT was used, e.g. p < 0.01) compared to voxel-wise inference in individual studies^32^, voxel-wise inference remains rather conservative for both individual studies and ALE^32,36^. For the sake of both sensitivity and specificity, the cluster-level FWE correction method is still regarded as the most appropriate method for statistical inference in ALE^36^. Therefore, we used cluster-level FWE correction method with a relatively more stringent CDT (p < 0.001) for a better control on the false-positive rates in our current ALE study. Under such setting, we did not find any consistent ASD-related resting-state local overconnectivity. This corroborates the recent conclusion that the hypothesis of ‘general local overconnectivity’ in ASD is likely to be unproven^26^. Such a conclusion was drawn mainly based on ReHo results, a technique which often attracts criticism on producing inconsistent regional findings due to the choices of motion correction, smoothness, GSR, and standardization^23,26^. We added to this conclusion by showing that the use of ICA, fALFF or ASL, in addition to ReHo, also did not produce any consistent overconnectivity patterns for ASD. Although studies using ABIDE database tend to report various local overconnectivity effects in the frontal and temporal cortices that seemingly replicate each other, for instance, in the superior temporal cortex^37,38^, and the dorsal superior frontal cortex or supplementary motor cortex^37–39^, such observations could plausibly be due to the inclusion of highly similar dataset from the same ABIDE pool. Alternatively, our meta-analysis study, which included some original datasets that have not been shared to the ABIDE pool at the time of our study, showed a different outcome. Although four of the included studies reported ASD-related local overconnectivity effects, no consistent effects could be obtained in our ALE analyses. Notably, the other four included studies also failed to detect any local overconnectivity effects. Our findings suggest that the mixed patterns of overconnectivity effects seen in some previous studies are more likely to be artifacts that differ substantially across studies and cannot be meaningfully unified in meta-analyses.

Under the same methodological considerations, two consistent ASD-related local underconnectivity effects were revealed in the dorsal PCC and right medial paracentral lobule. This suggests that the local underconnectivity effects are true effects that survive through the controversial preprocessing steps of analyzing resting-state data and the inclusion of ASD subjects with mixed characteristics. Being the first hub that exhibits the strongest connections of the default mode network in the developmental trajectory^40^, the PCC is involved in many important processes such as regulation and balancing the focus of attention to internal or external thoughts^41^, arousal and awareness^42^, and self-referential thought^43^. More specifically, it has recently been suggested that the ventral PCC may be responsible for internally directed cognition associated with the DMN such as planning for the future and memory retrieval, whereas the dorsal PCC may play a direct role in controlling our attentional focus by adjusting the stability of brain network over time (‘whole-brain metastability’)^44^. According to Leech and Sharp^44^, a high activity in the dorsal PCC supported increased whole-brain metastability for rapid transition between different cognitive processes in a broad attentional state. In contrast, a low activity in the dorsal PCC was related to a decrease in metastability, which allowed stable cognitive processing for a specific task in a narrow attentional state^44^. During resting state, a high dorsal PCC activity allows a broad attentional focus for the continuous flow of unconstrainted thoughts in our mind (‘freewheeling’). This state is associated with rapid transitions between neural states, and rapid changes of activity in intrinsic connectivity networks. Our results of local underconnectivity in the dorsal PCC suggest that a narrow attentional state may exist during resting state in subjects with ASD. According to the metastability model^44^, a narrow attentional state does not favor rapid transition between different cognitive processes. This corroborates with one of the hallmark features of ASD, that is, cognitive inflexibility^45^. Clinically, the level of cognitive inflexibility is measured by symptom severity of repetitive behaviors^46^, which was found to be negatively associated with the level of discriminability between task-evoked and resting brain states in children with ASD^47^. In other words, a weak modulation of brain states may underlie cognitive inflexibility in ASD^47^. This is in line with our finding of ASD-related resting-state underconnectivity in the dorsal PCC, which favors narrow attentional state during resting and does not support switching between brain states. Future studies should further explore whether the resting-state local connectivity of the dorsal PCC is correlated with the behavioral symptom of cognitive inflexibility in ASD, which may help explain the ‘need for sameness’ and an inability to flexibly adapt behavior in ASD.

According to behavioral domain meta-data of the BrainMap database, the dorsal PCC cluster was found to be engaged in a sub-domain of Emotion called ‘other’, which does not fall into any of the other Emotion sub-domains (e.g. intensity of emotion, valence of emotion, positive emotion such as happiness and negative emotion such as fear, anxiety and sadness)^35^. In other words, its activity cannot be solely attributed to the evaluations of the strength or aversiveness of an emotional stimulus, nor the two opposite ends of emotion. In fact, the ‘Emotion: other’ sub-domain is represented by several large clusters of activation spanning through the medial prefrontal cortex (PFC) to anterior and posterior cingulate cortices, as well as subcortical regions such as the insula, amygdalae, thalamus and caudate (see http://brainmap.org/taxonomy/behaviors/Emotion.html). These brain regions overlap with several components of the so-called ‘social brain’, for instance, the medial PFC, PCC and amygdala, which are important for affective aspects of social processing^48^. As the PCC is implicated in both the DMN and theory of mind network, it is believed that the PCC is crucial for supporting a common-sense understanding of social behavior to facilitate social interaction^48^. Notably, impaired social functioning and deficits in emotional understanding are persistent features of ASD according to the Diagnostic and Statistical Manual of Mental Disorders (DSM-V)^49^ and International Statistical Classification of Diseases and Related Health Problems (ICD-10)^50^. A recent study showed that the delayed development in emotional understanding in ASD was associated with severity of social problems^51^. Therefore, it is possible that our findings in the dorsal PCC may also be related to a weakened ability on interpreting social signals, especially those requiring affective processing, in people with ASD.

We also observed a lower resting-state local connectivity in the right medial paracentral lobule in subjects with ASD compared to TD controls. This abnormality was seen across adolescent^23,26^ and middle-aged samples with ASD^28^, suggesting that this abnormality could be persistent in ASD. The paracentral lobule is a continuation of the precentral gyrus in the frontal lobe and the postcentral gyrus in the parietal lobe towards the medial surface of the cerebral hemisphere. The observed medial paracentral cluster in this study belongs to the portion continued from the postcentral gyrus (i.e. the somatosensory cortex) as it locates posterior to the central sulcus^52^. This posterior portion plays a role in somatosensory processing of the lower limbs^53^, and the parietal lobe is involved in integrating sensory and somatosensory information from different body parts^54,55^. Our findings in the medial paracentral lobule may, therefore, be related to the impairment of multisensory integration which is commonly seen in ASD^56^. On the other hand, it is believed that the paracentral lobule works with the precuneus to generate the representation of the body in the spatial world, which is important for producing a sense of self^57^. An intact representation of the physical self appears to be important to the development of an abstract self^56^. Impairment to the paracentral lobule and its functional connections with other regions may therefore disrupt the representation of oneself in the world that further affects the development of theory of mind in ASD^58,59^. Future studies are warranted to confirm our speculations.

There are several limitations in this study. First, the sample size was relatively small in this meta-analysis, which may lead to a higher chance of committing Type I and Type II errors. When cluster level FWE correction method is applied, seventeen experiments were recommended to be included in an ALE meta-analysis to avoid results that are largely driven by one experiment^36^. For analyses involving less than ten experiments, the likelihood that results are largely driven by a singly experiment increases, especially if the experiment has a relatively larger sample size than the remaining studies^36^. In the current study, the findings of underconnectivity in the right medial paracentral lobule cluster and the dorsal PCC cluster were contributed by three studies^23,26,28^ and two studies^23,26^, respectively. None of these contributing studies had a substantially larger sample size than the remaining ones, suggesting the chance of having a dominant effect from a singly study is low. To further confirm the finding in the PCC, we did a narrative review from three excluded studies that examined only the DMN resting-state abnormalities in ASD. In line with our findings, two studies demonstrated hypoconnectivities in the PCC in adults (PCC coordinates in Talairach space: x = −5, y = −49, z = 40)^60^ and teenagers (PCC coordinates in Talairach space: x = −3, y = −40, z = 13)^61^ with ASD. The other study reported reduced resting-state functional connectivity in DMN regions including the PCC (coordinates were not provided) in children (1–8 years old) with ASD compared with TD controls^62^, indicating that the abnormalities that we found might also be observed in children with ASD. To confirm our findings and explore other neural abnormalities in ASD with a greater power, another comprehensive meta-analysis of the same topic with a larger sample (e.g. twenty experiments^36^) shall be conducted in future when more relevant studies are available. Last, although follow-up behavioral analyses were performed for the right medial paracentral lobule cluster and the dorsal PCC cluster, the association between neural markers and behavioral symptoms in ASD cannot be deduced using this ALE method. Despite the limitations, our findings demonstrated two ASD-related resting-state local underconnectivity in the dorsal PCC and right medial paracentral lobule, which are consistent across studies with mixed subject characteristics and methodology. Findings from behavioral analyses support the role of the dorsal PCC in weakened ability on interpreting social affective signals, and we speculate that it might also link to cognitive inflexibility. The right medial paracentral finding may be associated with the altered representation of the physical self in ASD, which seems to be common across ASD subjects of different ages. Overall, this study contributes to uncovering a consistent pattern of resting-state local abnormalities in ASD, which may serve as potential neurobiological markers for ASD. Future studies should examine the specificity and sensitivity of detecting these abnormalities for early identification of people who are vulnerable to ASD.

## Methods

A comprehensive online literature search on the MEDLINE/PubMed databases was conducted, focusing on functional neuroimaging studies on ASD. Keyword searches were conducted using the following search terms: (1) “neuroimaging” <OR >“fMRI,” (2) “resting state” <OR >“default network” and (3) “autism spectrum disorder” <OR >“ASD” <OR >“autism”. These searches were confined to articles published in English up to February 2018, which yielded 278 original or review articles. We also searched through the reference list of relevant review articles for additional studies. From these research articles, we included studies that reported Montreal Neurological Institute (MNI) or Talairach coordinates of whole-brain contrast comparing ASD subjects and TD healthy controls. Two of the authors (WKWL and MKL) confirmed the inclusions of the identified studies. Studies were excluded if (1) no control group was included; (2) they were review articles; (3) only *a priori* region of interest (ROI) analysis, seed-based functional connectivity analysis or graph theory analysis was conducted; (4) independent component analysis (ICA) was performed and only the default mode network (DMN) component or another specific component was examined; (5) only task-based fMRI experiment was reported; or (6) only online fMRI datasets such as ABIDE, were used. According to the recommendation from the BrainMap team (see http://brainmap.org/taxonomy/criteria.html), studies that intentionally restrict their analyses to a ROI (e.g. the DMN component) that is substantially smaller than the whole brain should be excluded. Otherwise, the ALE algorithm may be biased to report the foci from the ROI studies as significant, because it assumes that each effect in the brain is approximately equally likely to occur, resulting in an increase in false positive rate. The exclusion of online dataset is to ensure that our ALE analyses will not be falsefully biased by including similar results generated from the same data source. Nine studies met our inclusion criteria, which included eight fMRI studies and 1 arterial spin labeling (ASL) study. Among the eight fMRI studies, three of them used regional homogeneity (ReHo)^23,24,26^, one of them used fractional amplitude of low frequency fluctuations (fALFF)^30^, and four of them used ICA^27–29,31^. One of the studies did not find any spatial group difference^31^, therefore, it was not included in the ALE meta-analysis. Eight studies (eight experiments) involving 424 subjects were finally included in the ALE meta-analysis (Table 1). All of them reported reduced resting-state local connectivity in ASD compared to TD controls (eight experiments, 28 foci). Four of them found increased resting-state local connectivity in ASD compared to TD controls (4 experiments, 18 foci) (Table 1).

The coordinate-based ALE analysis was conducted by GingerALE version 2.3.6 (The BrainMap Database, www.brainmap.org; San Antonio, TX, USA). Coordinates in MNI space were imported into the software. Imported foci were modeled as three-dimensional Gaussian spatial probability distributions using a full-width at half-maximum (FWHM) kernel estimated based on the corresponding experiment’s sample size^63^. The ‘non-additive’ method was used to combine these probability distributions and generate a modeled activation map^64^. The union of the modeled activation maps of each experiment was then created to form the ALE image. The ALE image contains the combined probability distribution of finding an activation being located at that particular voxel, which is the ALE score. To better control for the false-positive rates, the ALE image was then thresholded using uncorrected *p* < 0.001 and a cluster-level inference threshold of *p* < 0.05 with 5000 permutations of simulated random data based on the characteristics of the imported data^65^.

Group differences in resting-state local connectivity among the ASD and TD participants were examined using the results of between-group contrasts (ASD < TD and ASD > TD) from individual studies. Since the study conducted by Nair and colleagues^26^ reported three sets of results using different analysis procedures (standardized ReHo with the use of global signal regression, GSR; standardized ReHo without using GSR; nonstandardized ReHo without using GSR; see Table 1^d,e,f^), three separate ALE analyses were performed for each of the between-group contrast by including one of the three sets of their results each time. As to the study conducted by Maximo and colleagues^23^, according to the authors, the results of applying GSR and not applying GSR, and the results of applying standardization and not applying standardization, were highly similar. Therefore, we did not separately test for the different sets of results of their study. As a result, a total of six ALE analyses were performed.
